# The Relationship Between Weight Self-Stigma and Quality of Life Among Youth in the Jazan Region, Saudi Arabia

**DOI:** 10.7759/cureus.18158

**Published:** 2021-09-21

**Authors:** Bayan H Khodari, Mohammed O Shami, Reem M Shajry, Joudi A Shami, Abdellh A Names, Afnan A Alamer, Azhar M Moafa, Reem O Hakami, Ghaida S Almuhaysin, Ahmad Y Alqassim

**Affiliations:** 1 Family Medicine, Ministry of Health Holdings, Jazan, SAU; 2 College of Medicine, Jazan University, Jazan, SAU; 3 Cardiology, Jazan University, Jazan, SAU; 4 Family and Community Medicine Department, Faculty of Medicine, Jazan University, Jazan, SAU

**Keywords:** saudi arabia, public health, environmental health, youth, quality of life, weight self-stigma

## Abstract

Background: Studies on the role of weight self-stigma on the quality of life of young adults are limited. Therefore, the present study aimed to examine the relationship between different forms of weight self-stigma (self-devaluation and fear of enacted stigma) and the quality of life among Saudi youth in the Jazan region.

Methods: A cross-sectional study was performed using a sample of 399 participants who were invited through social media platforms. We used Arabic, validated versions of the weight self-stigma questionnaire (WSSQ) and the World Health Organization quality of life questionnaire. Data analysis was performed by independent samples t-test and analysis of variance with Tukey's post hoc test.

Results: The study sample consisted of 399 participants aged 21.12 years ± 2.91 years. A total of 264 (66.2%) were female. The overall weight self-stigma score of the study population was 34.81 ± 10.73 on the WSSQ. The analysis showed a significant association between weight self-stigma and quality of life and body mass index (BMI; p < 0.01). In addition, participants who were overweight and obese had more self-devaluation and fear of enacted stigma than did participants with normal weight. Participants with high weight self-stigma had lower quality of life than did those with less weight self-stigma.

Conclusions: The results of this study show that weight self-stigma is negatively correlated with the individual's quality of life. Weight self-stigma was positively associated with BMI.

## Introduction

There are two sorts of stigma: public stigma and self-stigma. Self-stigma is defined as awareness of and agreement with public stigma stereotypes and attitudes and the application of those stereotypes to oneself, which undermines self-esteem and self-efficacy [1]. Weight self-stigma is defined as personal experiences of shame, negative self-evaluations, and perceived discrimination [2]. Weight stigma is a state of social devaluation of people with overweight or obesity. It often includes negative associations and stereotypes expressed differently, leading to prejudice, social exclusion, blatant unfair treatment, negative stereotypes, and discrimination [3]. Weight stigma can be divided into two types: perceived weight stigma and internalized weight stigma. According to WHO, obesity and overweight are described as abnormal or excessive fat accumulation that presents a health risk. Being overweight is considered a BMI of 25 or higher, and obesity is considered a BMI of 30 or higher [4]. Obesity and overweight among children and adolescents are becoming increasingly prevalent public health issues in many parts of the world [5,6]. The overweight and obesity, according to WHO criteria in Saudi Arabia, has a high prevalence, especially among Saudi females [7]. The effect of weight stigma on psychological, social, and academic outcomes in youth has been linked to different physical and mental conditions such as depression, low self-esteem, physiological stress, unhealthy eating, body dissatisfaction, suicidal behavior, poor academic performance, increased obesity, and diabetes [8]. In addition, according to studies, weight discrimination is expected and may be a daily occurrence for people who are overweight or obese [9]. Weight stigma can lead to overeating, social isolation, underutilization of healthcare services, decreased physical activity, and weight gain, worsening obesity and posing additional barriers to healthy behavior change [10]. For example, a landmark study showed that adolescents with severe obesity were more likely to suffer weight stigma and had lower quality of life scores than their peers with cancer [11].

Furthermore, BMI was significantly inversely associated with physical, social, and psychosocial functioning in children and adolescents [11]. Additionally, bias and discrimination are not limited to older teens, and youngsters were subjected to negative weight stereotypes [12]. Adolescence is a critical developmental stage marked by independent growth, physiological changes, academic demands, and new experiences [13]. According to a study on adolescents seeking weight-loss treatment, 71% said they had been bullied for their weight problems in the previous year, and more than a third said the bullying had gone on for five years or longer [10]. Weight stigma can harm young people's quality of life [14]. Weight stigma can lead to overeating, social isolation, underuse healthcare facilities, decreased physical activity, and weight gain, increasing obesity and creating additional barriers to healthy behavior changes [14]. The seminal study by Schwimmer et al. [11] also found that children and adolescents with extreme obesity were more likely to experience weight stigma and had lower quality of life ratings than their cancer-free peers.

Furthermore, racism and discrimination are not limited to older teenagers. Children's negative weight stereotypes began as early as the age of three [12]. Therefore, the main objective of this study is to study the relationship between weight self-stigma and quality of life among youth in the Jazan region.

## Materials and methods

Study design and participants

A cross-sectional, observational study was conducted between August 3 and September 1, 2021. A convenient random sample of 440 participants was calculated based on a confidence interval of 95%, error not exceeding 5%, nonresponse rate of 20%, and the following sample size formula:

\ n =\frac{Nz_{(\alpha)}^2 P (1 &minus; P)}{(N &minus; 1)d^2+ P(1 &minus; P)z^2_{(\alpha) }}\\

The participants were invited to fill an online survey distributed through social media websites and applications (WhatsApp, Facebook, Twitter, Snapchat). A self-report questionnaire was designed by Google forms and made available throughout the study to allow anonymous, voluntary participation. The inclusion criteria were Arabic-speaking young adults (those persons between the ages of 15 and 24 years) [15] residing in the Jazan region, Saudi Arabia, at that time.

Measures

Socio-Demographic Variables

In the first section, the survey included questions about participants' demographics like age, sex, marital status, employment status, education level, monthly income, and area of residency.

Weight Self-Stigma Questionnaire

The second section consisted of the Arabic version of the weight self-stigma questionnaire (WSSQ), which is a valid and reliable indicator composed of a 12-item Likert-type scale of weight-related self-stigmatization [16]. It has two subscales that measure weight-related self-devaluation and fear of enacted stigma. The Arabic WSSQ has good internal consistency and reliability. Internal consistency was α = 0.898 for the overall survey, and the test-retest reliability of the intraclass correlation coefficient was α = 0.982. The factorial structure is similar to the original English WSSQ, which endorses its value in cross-cultural studies [16]. Our analysis showed an internal consistency of α = 0.840 for the self-devaluation domain of the WSSQ and 0.876 for the fear of enacted stigma domain. Therefore, the Arabic WSSQ appears to be a reliable measure for assessing weight-related self-stigma in Arabic-speaking people.

The World Health Organization Quality of Life Instrument

The fourth section measures the quality of life using an Arabic translation of the World Health Organization Quality of Life (WHOQOL)-BREF, which the WHO designed to measure the quality of life [17,18]. The WHOQOL-BREF is a cross-culturally applicable version of the WHOQOL-100 developed by the WHOQOL Group30 to assess the quality of life in many aspects [19]. It includes 26 items organized into four domains: physical health (seven items), psychological health (six items), social relationships (three items), and environment (eight items), as well as a self-rating of the general quality of life (one item) and general satisfaction with health (one item). It is self-administered, and each item is scored on a range of 1 to 5, with higher scores indicating a better quality of life. In addition, each domain score (mean score of items within that domain) is transformed to a 0-100 scale representing an individual's impression of that domain's quality of life. Our analysis showed an internal consistency of α = 0.795 for the physical domain, 0.811 for the psychological domain, 0.685 for the social domain, and 0.819 for the environmental domain.

Statistical analysis

After data collection, data were verified manually and then coded within an excel sheet. Statistical analysis was conducted using the Statistical Package for the Social Sciences (SPSS version 25, IBM Corp., Armonk, NY). Data were analyzed using descriptive and comparative statistics. Descriptive statistics were calculated for study variables, e.g., frequency and percentage for qualitative variables and mean and standard deviation for quantitative variables. Associations between variables were calculated using independent samples t-test or one-way analysis of variance (ANOVA) as appropriate. Post-hoc Tukey's honest significant difference (HSD) was performed to examine significant associations in detail. Values of p < 0.01 or 0.05 were considered statistically significant.

Ethical consideration

The study had been ethically approved by Jazan Health Ethics Committee (approval number 2157, dated August 03, 2021). Minor participants (less than 18 years old) were asked for parental consent before participation. All procedures performed in our study involving human participants were in accordance with the ethical standards of the institutional and/or national research committee and with the 1964 Helsinki declaration and its later amendments or comparable ethical standards.

## Results

Baseline characteristics of the sample

Table 1 shows a detailed description of the sample baseline characteristics. Among the 399 participants who agreed to participate in this survey, 264 (66.2%) were female. The mean age was 21.12 years ± 2.91 years. The highest education level obtained was bachelor's degree in 258 (64.5%) subjects, high school degree in 126 (31.5%) subjects, and middle school degree in 15 (3.8%) subjects. Monthly income (in Saudi Riyals) was more than 15,000 in 133 (33.33%) of the study population. Almost half of the participants lived in villages (N = 195, 48.8%). BMI calculation showed a considerable proportion (N = 92, 23.1%) of subjects with overweight scores, 29 (7.3%) with obesity class I, 19 (4.8%) with obesity class II, and 18 (4.5%) with obesity class III (Table 1).

**Table 1:** Baseline characteristics of the sample. N: frequency; M: mean; SD: standard deviation; BMI: body mass index.

Characteristics	N	%
Age in years, M (SD)	21.12 (2.91)	
Sex		
Male	135	33.8
Female	264	66.2
Education		
Middle school	15	3.8
High school	126	31.5
Bachelor	258	64.5
Monthly income (Saudi Riyals)		
0−4999	101	25.3
5,000−9,999	84	21.1
10,000−15,000	81	20.3
>15,000	133	33.3
Residency location		
City	195	48.8
Village	194	48.5
Mountain areas	10	2.5
BMI class		
Underweight (BMI < 18.5 kg/m^2^)	69	17.3
Normal weight (18.5 ≤ BMI < 25.0 kg/m^2^)	168	42.1
Overweight (25.0 ≤ BMI < 30.0 kg/m^2^)	92	23.1
Obese I (30.0 ≤ BMI < 34.5 kg/m^2^)	29	7.3
Obese II (35.0 ≤ BMI < 39.5 kg/m^2^)	19	4.8
Obese III (BMI ≥ 40.0 kg/m^2^)	18	4.5

Prevalence of weight self-stigma

Because the WSSQ suggests no cut-off scores to dichotomize responses, the analysis shown in Table 2 explores the prevalence of individual items of the questionnaire among the study population. On average, subjects scored 34.81 ± 10.73 on the comprehensive questionnaire of weight self-stigma. For self-devaluation statements, the highest prevalence of weight self-stigma was observed among subjects who reported a sense of inferiority (57.6%), followed by those who reported self-blame (52.1%), poor self-control (42.4%), guilt (41.9%), and weakness (32.8%). For fear of enacted stigma statements, 41.1% reported perceived lack of others' sympathy, 38.1% thought that others blame them for weight problems, 32.6% thought others believed that they lacked self-control because they did not control their weight, 20.1% reported enacted stigma from discrimination, and 16.5% reported a sense of shame.

**Table 2:** The weight self-stigma questionnaire. WSS: weight self-stigma. *Responses to the weight self-stigma questionnaire were recoded and summarized into positive (agree and completely agree), neutral, and negative (disagree and completely disagree) responses.

Items	Responses^*^					
	Disagree		Neutral		Agree	
WSS-total score, M (SD)	34.81 (10.73)					
Self-devaluation	N	%	N	%	N	%
I will always go back to being overweight	174	43.6	111	27.8	114	28.6
I caused my weight problems	100	25.1	91	22.8	208	52.1
I feel guilty because of my weight problems	148	37.1	84	21.1	167	41.9
I became overweight because I am a weak person	181	45.4	87	21.8	131	32.8
I would never have any problems with a weight if I was stronger	96	24.1	73	18.3	230	57.6
I do not have enough self-control to maintain a healthy weight	130	32.6	100	25.1	169	42.4
Fear of enacted stigma						
People discriminate against me because I have had weight problems	238	59.6	81	20.3	80	20.1
It is difficult for people who have not had weight problems to relate to me	137	34.3	98	24.6	164	41.1
Others will think I lack self-control because of my weight problems	169	42.4	100	25.1	130	32.6
People think that I am to blame for my weight problems	163	40.9	84	21.1	152	38.1
Others are ashamed to be around me because of my weight	268	67.2	65	16.3	66	16.5
I feel insecure about others’ opinions of me	192	48.1	79	19.8	128	32.1

Self-devaluation

The mean and standard deviation of the respondents' scores on the weight self-stigma questions are summarized in Table 3. The scores for self-devaluation were higher (M = 21.92, SD = 5.25) among subjects with obesity (BMI ≥ 40.0 kg/m^2^) compared to subjects with normal weight (M = 17.62, SD = 5.07), underweight subjects, or overweight subjects (M = 20.62, SD = 5.06) (M = 15.30, SD = 6.17). A one-way between-subjects ANOVA was conducted to compare the effect of body weight on the level of self-devaluation. A significant difference was observed between subjects with different body weights, F (3, 391) = 23.86, p = 0.000. Post hoc analyses using the Tukey HSD test indicated that the mean scores for the self-devaluation domain were higher among obese subjects than subjects with normal weight, underweight, or overweight (Table 3). An independent-samples t-test indicated that scores for self-devaluation did not differ significantly between males and females, t(397) = 0.04, p = 0.060 (Table 3). A one-way between-subjects ANOVA found no association between self-devaluation and education level, F (2, 396) = 0.62, p = 0.540, or monthly income, F(3, 395) = 1.20, p = 0.311 (Table 3).

Fear of enacted stigma

The scores for the fear of enacted stigma were higher (M = 19.18, SD = 6.57) among subjects with obesity (BMI ≥ 40.0 kg/m^2^) compared to subjects with normal weight (M = 14.57, SD = 5.85), overweight subjects (M = 17.59, SD = 5.59), or underweight subjects (M = 15.41, SD = 6.35). A one-way between-subjects ANOVA was conducted to compare the effect of body weight on the level of fear of enacted stigma. A significant difference was observed between subjects with different body weights, F (3, 391) = 11.19, p = 0.000. Post hoc analyses using the Tukey HSD test indicated that the mean scores for the self-devaluation domain were higher among obese subjects than subjects with normal weight, underweight, or overweight (Table 3).

**Table 3:** Comparing weight-related self-stigma in the study population in terms of BMI, and sex type. DASS 21: Depression, Anxiety, and Stress Scale 21; M: mean; SD: standard deviation; BMI: body mass index; SAR: Saudi Riyals. Independent samples t-test or one-way ANOVA used as appropriate. Post-hoc Tukey’s HSD test is as follows: ^a^p < 0.01 vs normal weight. ^b^p < 0.01 vs overweight. ^c^p < 0.05 vs normal weight. *p < 0.05; **p < 0.01.

Variables/categories		Self-devaluation			Fear of enacted stigma		
		M	SD	F or t	M	SD	F or t
All subjects		18.65	5.74		16.16	6.35	
BMI class	Underweight	15.30^bc^	6.17	23.86^**^	15.41^a^	6.89	11.19^**^
	Normal weight	17.62^b^	5.07		14.57^b^	5.85	
	Overweight	20.62^a^	5.06		17.59^a^	5.59	
	Obese	21.92^a^	5.25		19.18^a^	6.57	
Sex	Male	18.64	5.76	0.04	15.33	6.17	1.89
	Female	18.66	5.74		16.59	6.40	
Education	Middle school	20.27	7.67	0.62	16.20	6.38	0.49
	High school	18.57	5.80		16.62	6.82	
	Bachelor	18.60	5.59		15.94	6.12	
Monthly income (SAR)	0−4999	18.52	6.43	1.20	16.68	7.30	0.51
	5,000−9,999	18.71	5.56		15.52	5.63	
	10,000−15,000	19.63	5.07		16.23	5.57	
	>15,000	18.11	5.66		16.13	6.48	

An independent-samples t-test indicated that scores for self-devaluation did not differ significantly between males and females, t(397) = 1.89, p = 0.971 (Table 3). A one-way between-subjects ANOVA found no association between fear of enacted stigma and education level, F (2, 396) = 0.49, p = 0.615, or monthly income, F (3, 395) = 0.51, p = 0.674.

Quality of life and psychological distress

Table 4 shows the correlation between weight self-stigma and quality of life. The means of each subscale of the DASS 21 were described in Table 3. Weight self-stigma correlated negatively with physical health [r(397) = 0.27, p = 0.000], psychological health [r(397) = 0.34, p = 0.000], social relationships [r(397) = 0.20, p = 0.000], and environmental health [r(397) = 0.31, p = 0.000]. This means that the quality of life was significantly reduced in subjects who reported weight self-stigma (Table 4).

**Table 4:** Correlation between weight self-stigma and quality of life. M: mean; SD: standard deviation; r: Pearson correlation coefficient. ** p < 0.01.

		M	SD	Weight self-stigma
Quality of life	Physical domain	64.46	18.82	−0.27^**^
	Psychological domain	82.21	19.90	−0.34^**^
	Social domain	84.37	27.06	−0.20^**^
	Environmental	84.07	19.03	−0.31^**^

## Discussion

Weight-related stigmatization has debilitating consequences that arise from the prevailing negative attitudes toward obese patients and lead to discrimination in several aspects of life, including employment, education, relationships, and healthcare [20]. The present study contributes to understanding these consequences, focusing on the effect of weight self-stigma on the quality of life in Saudi young adults.

As expected, the prevalence of weight-self stigma among the surveyed subjects was high. On average, the mean score of the overall weight self-stigma was 34.81 ± 10.73, demonstrating that a recognizable number of young adults enrolled in this survey had symptoms of weight self-stigma (the minimum score for the weight self-stigma questionnaire is 12). High total weight self-stigma among adults has been demonstrated by similar studies using the same assessment tool. In a study by Farhangi et al. [21], the total weight self-stigma was 31.44 ± 10.00, which is comparable with what was found in the present study. Interestingly, the relationship between self-devaluation and fear of enacted stigma showed a consistent trend that weight self-stigma was positively proportional to the individuals' BMI. Previous studies provided strong evidence that suffering weight stigma is more likely with increasing BMI [22]. However, other studies produced heterogeneous results regarding the association between weight stigma and BMI. While some authors demonstrated a positive correlation between internalized weight stigma and BMI [23], others found no association between the internalization of weight bias and degree of overweight [24]. The prevalence of overweight in Saudi Arabia among adult males and females was 33.4% and 33.4%, respectively, while 24.1% adult males and 33.4% females were overweight [25]. As we note, being overweight or obese is not uncommon in Saudi Arabia. Therefore, when we talk about weight self-stigma toward a third of society, it seems to be an exceptional phenomenon. Although, our result showed that a large portion of participants confirms the existence of weight self-stigma.

Weight stigma showed moderate and negative correlation with the physical, psychological, social, and environmental aspects of quality of life in this population. Such a result agrees with previous findings that weight-related stigma is associated with lower physical [26] and psychological functioning [27] in overweight and obese adults. Higher psychological stress associated with weight self-stigma plays a critical role in the diminished quality of life of affected individuals. It is well known that obese patients with weight self-stigma have an increased risk of psychological stress. A large body of research has shown that individuals with overweight and obesity are perceived negatively and provoke significantly more feelings of disgust than homeless and mentally ill people, among other historically stigmatized groups [28]. The psychological domain in our study showed the strongest correlation with high weight self-stigma. Perceived stress in individuals with fear of enacted stigma can contribute to cognitive impairment and poor socialization [29]. This is reflected in the finding of reduced social health among people with weight stigma in this study. Furthermore, psychological stress was a vital risk factor for mediating the relationship between weight stigma and eating disorders in a multivariate model adjusting for age, gender, and BMI [30].

Strengths and limitations

The current study was valuable as it is the first study examining the prevalence of weight self-stigma and its role in the quality of life of Saudi young adults using validated and reliable assessment tools. However, the study had several limitations. The causal relationship between weight self-stigma and other variables could not be established, given the study's cross-sectional design. Although the survey was equally distributed among the target population, female respondents constituted two-thirds (66.2%) of the study sample. An unequal sex ratio may limit the generalizability of our findings.

## Conclusions

Our findings demonstrated that weight self-stigma was strongly associated with BMI and negatively correlated with the quality of life of overweight and obese individuals. Furthermore, these findings show that weight-related stigmatization is a health concern in Western countries and Middle eastern developing countries like Saudi Arabia. This is possible due to the dispersion of Western stigmatization norms through urbanization and social media. Therefore, interventional strategies at the individual and societal levels should target the patient's weight self-stigma by improving coping skills with negative consequences of weight-related stigma to increase their quality of life.
